# The Hammer and the Nail: Biomechanics of Striking and Struck Canadian University Football Players

**DOI:** 10.1007/s10439-021-02773-4

**Published:** 2021-04-23

**Authors:** Jeffrey S. Brooks, Adam Redgrift, Allen A. Champagne, James P. Dickey

**Affiliations:** 1 School of Kinesiology, Faculty of Health Sciences, Western University, 1151 Richmond St., London, ON Canada; 2 Centre for Neuroscience Studies, Queen’s University, Kingston, ON Canada; 3 School of Medicine, Queen’s University, Kingston, ON Canada

**Keywords:** Concussion, Linear, Rotational, Acceleration, Head impacts, Impact biomechanics, Injury prevention, Football, Sub-concussive impacts

## Abstract

This study sought to evaluate head accelerations in both players involved in a football collision. Players on two opposing Canadian university teams were equipped with helmet mounted sensors during one game per season, for two consecutive seasons. A total of 276 collisions between 58 instrumented players were identified via video and cross-referenced with sensor timestamps. Player involvement (striking and struck), impact type (block or tackle), head impact location (front, back, left and right), and play type were recorded from video footage. While struck players did not experience significantly different linear or rotational accelerations between any play types, striking players had the highest linear and rotational head accelerations during kickoff plays (*p* ≤ .03). Striking players also experienced greater linear and rotational head accelerations than struck players during kickoff plays (*p* = .001). However, struck players experienced greater linear and rotational accelerations than striking players during kick return plays (*p* ≤ .008). Other studies have established that the more severe the head impact, the greater risk for injury to the brain. This paper’s results highlight that kickoff play rule changes, as implemented in American college football, would decrease head impact exposure of Canadian university football athletes and make the game safer.

## Introduction

In recent years, biomechanical studies of head impacts in football players have enabled scientists to gather more insight about the mechanisms of injury as a way to better understand and improve prevention strategies of sport-related concussions.^38^ Injury thresholds and cumulative impact exposure risk have commonly been assessed, with difficulty defining an injury-specific threshold,^22^ probability percentage for concussion,^50^ incidence predictions based on position^20^ and session,^44^ and the yet unknown association between the cumulative effects^18^ of such impacts over time, throughout an athlete’s career. Many studies have shifted their focus to quantifying head impact exposures by collecting head impact data from football players over extended periods of time.^6^,^10^,^15^,^16^ While the purpose and results of these studies vary, a common understanding is that the more severe the head impact, the greater risk for injury to the brain.^23^,^35^,^50^ In order to better understand the mechanism of injury, some studies have also focused on individual impacts. This provides a more individualized framework that may account for the heterogeneity in biomechanical factors that relate to colliding athletes.^10^ Thus, a thorough identification of the plays and parts of the football game that are associated with more severe head impact magnitudes is essential to minimize risk of head injury in football players.^13^,^36^,^38^

Concussions in the NFL have gained national and international attention, and have recently been studied using videogrammetry.^3^ Attempts at reconstructing professional football impacts in the lab have reported average head kinematics for striking and struck players,^45^,^46^ although these included a small sample of reconstructed videos (*n* = 27) and focused on impacts that resulted in concussive injury. Furthermore, it is worth noting that only helmet-to-helmet collisions were evaluated, and that none of the striking players suffered concussions in these impacts. This in turn constrains the generalizability of those results, providing limited information regarding the kinematics of non-concussive head impacts between colliding football players. Other studies have employed finite element modelling to determine brain strains from laboratory head impact reconstructions.^19^,^21^,^51^ These studies determined that head impact location has a large effect on regional brain strain, emphasizing the need to account for such parameters when modelling head impact strain forces on the brain.^19^ Additionally, these studies of reconstructed impacts suggest possible differences between players delivering the impact and receiving the impact, as well as the influence of head impact location on brain injury, inviting such analysis to be conducted on collegiate athletes.

In contrast to laboratory reconstructions, it is relatively straightforward to collect large data sets of actual football head impacts in games using wearable sensors. A college football study instrumented football players with sensors and determined that player anticipation did not affect head impact severity, and that struck players experience greater rotational accelerations compared to striking players.^30^ In agreement with the finite element modelling studies, they also determined that impact location affected head impact severity for striking and struck players. However, this study only evaluated one of the players in each impact event – either the striking or the struck player. The researchers acknowledged that head impact measurements may differ if both players involved in a collision were measured. Despite those findings, no studies have characterized the kinematics of impacts between two instrumented players, within a competitive setting, where the risk for head injury is higher.^24^, ^38^

**Figure 1:**
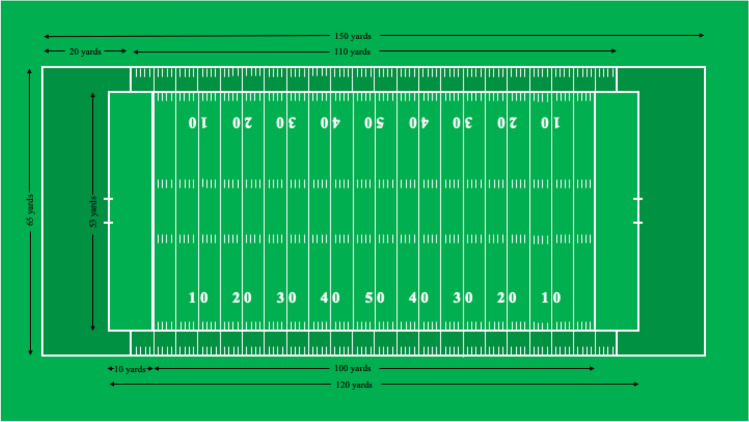
Illustration of an American sized field over top of a Canadian field.

All previously mentioned studies examine American football. The Canadian game of football has several rules that set it apart from the American game and could influence head impact magnitudes. The field size is larger (Fig. 1; CAN = 110 x 65 yds, US = 100 x 53yds), players can be in motion before the snap of the ball, there is one fewer attempt to achieve a first down (CAN = 3, US = 4), and there is one more player on the field for each team (CAN = 12, US = 11). The larger field size and players in motion may result in larger head impact magnitudes due to a potential for larger closing distance between the striking and struck player.^34^ As well, due to the fewer number of downs and more players on the field, the Canadian game typically involves more passing plays; a pass-style offensive scheme is associated with higher magnitude head accelerations than a run-style scheme.^29^ Additionally, the fewer number of downs results in more special teams plays, where higher magnitude impacts occur than on offensive or defensive plays.^34^ Accordingly, it is important to evaluate the magnitude of head impacts in Canadian football as American data cannot be generalized to the Canadian game.^6^^–^8,31

The purpose of this study was to characterize kinematic head impact magnitudes between instrumented striking and struck Canadian university football players. We hypothesized that struck players would experience higher head impact magnitudes than striking players, tackling collisions would result in larger head impact magnitudes than blocking collisions, head impact magnitudes would vary by location on the head, and that special teams plays would experience higher head impact magnitudes than offensive or defensive plays.

## Materials and Methods

Participants: Select members of two Canadian university football teams that were part of larger studies at each location were eligible. Other components of these studies have been published.^6^,^11^,^12^ This study was approved by both local research ethics boards, and all participants provided informed consent. The two teams faced each other once during each Fall USports regular season of play in 2017 and 2018. A total of 156 unique players competed in these games, 94 of whom were equipped with a helmet-mounted sensor. The participants in this study had to have experienced a head impact with an opposing player, and both of the players had to be equipped with sensors. All impacts were verified on video to establish a ground truth dataset, a suggested best practice for helmet-mounted head impact sensors.^14^,^32^,^49^

**Figure 2:**
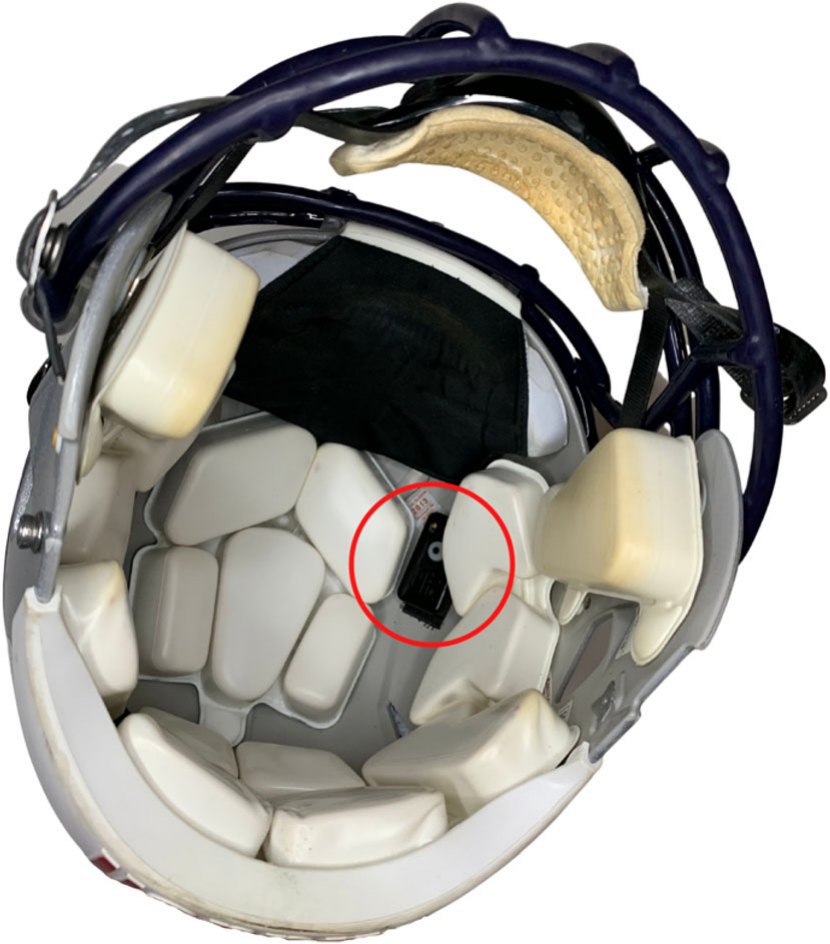
GForce Tracker, circled in red, attached to the inside of a Riddell Speed helmet, right of the crown cushion, using an industrial-strength recloseable fastener.

Helmet Instrumentation: The GForce Tracker (GFT) was used by both teams to measure helmet impacts (Artaflex Inc., Markham, ON, Canada). One GFT was attached to the inside of each participant’s helmet, right of the crown cushion, using an industrial-strength recloseable fastener (Fig. 2; 3M™ Dual Lock™ Recloseable Fastener SJ3551 400 Black, St. Paul, MN). Previous studies have used^4^,^7^,^9^,^12^,^17^ and validated^9^ this location and mounting. The GFT triggered when the helmet linear acceleration exceeded the user-defined threshold. This study used a threshold of 15 *g,* which is consistent with best practices.^26^ Each impact was time stamped and recorded to the device’s onboard storage.

Impact Data Protocol: The GFT data were transferred to a laptop after each game and then uploaded to GForce Tracker’s cloud-based storage. Summary files describing every impact (time stamp, peak linear acceleration, peak rotational velocity, and helmet location) were later downloaded for analysis.

Data reduction extracted the peak linear acceleration and peak rotational velocity and acceleration for each head impact. Similar to previous research,^9^ the peak resultant linear acceleration and peak resultant rotational velocity and acceleration at the centre of mass of the head were estimated using a correction algorithm based on impact location dependent equations. Since the accelerations at the centre of mass of the head were lower than at the helmet shell, the correction algorithm effectively attenuated the peak linear acceleration and rotational velocity and acceleration.

Video Data Protocol: Game video was recorded and analyzed using a Sony Vixia HD camera (EVS25, Endzone Video Systems, Sealy, Texas, United States). Game time and time of day were recorded for each game to match sensor time stamps to game video. Each game was uploaded to a video analysis software program (dba HUDL, Agile Sports Technologies Inc., Lincoln, Nebraska, United States). The game videos from both seasons were reviewed by one of the authors to verify every impact used in the analysis using the video software tool.

Only head impacts between players instrumented with helmet sensors were analyzed. Head impacts were first identified via video and confirmed with matching helmet sensor time stamps. Each collision between two players was given a unique identifier to associate impacts between specific pairs of players. Each impact was classified according to the player, play type, impact type, player involvement, opposing player impacted, and position by a single rater using a standardized rubric created for this study. Player positions were defined as defensive backs, linebackers, defensive and offensive linemen, running backs, quarterbacks, and wide receivers. Impact type was either tackle or block. Play type consisted of pass and run for offensive and defensive plays, and field goal, punt, punt return, kickoff, and kick return for special teams plays. Player involvement categorized impacts into striking or struck actions. A player was classified as striking if they initiated the collision with their opponent. A player was classified as struck if they were contacted by an opposing player. During impact observations, the rater was blinded to the head impact kinematic data.

Statistical Analysis: A Shapiro-Wilks test was used to determine the normality of the head impact magnitude distributions. Normally distributed parameters are reported as mean and standard deviation, and non-normally distributed parameters are reported as median and interquartile range. Means and standard deviations are reported for the linear mixed effects model as these models are robust to non-normally distributed data. Age, mass, and height of participants were measured at the start of the football season.

All statistical analyses were performed in R,^37^ with linear mixed effects analyses conducted using the lme^45^ and lmerTest^27^ packages. Three linear mixed effects models were created. One evaluated linear acceleration, one evaluated rotational velocity and the other evaluated rotational acceleration. The fixed effect of player involvement separately interacted with the fixed effects of impact type, game scenario, and impact location within both the linear and rotational models. Random effects of players involved in each collision were included in all models to account for player and positional differences across both teams. Impact locations were back, front, left and right on the helmet. Treatment contrasts were used to compare each level of fixed effect to the reference level.

Post-hoc analyses were conducted using Tukey multiple comparison tests from the emmeans package.^28^ Statistical significance was defined using a threshold of .05. Effect sizes in linear mixed effect modelling can be misleading and inaccurate,^5^ and therefore were not calculated.

## Results

Head impact data were collected from 58 players [age: 21.9 (1.7) years, mass: 100.8 (17.5) kg, height: 186.0 (5.6) cm], including defensive backs (n = 11), linebackers (n = 14), defensive (n = 10) and offensive linemen (n = 7), running backs (n = 8), quarterbacks (n = 1) and wide receivers (n = 7), representing 21 players from one team and 37 players from the other team. A total of 1085 impacts were recorded via helmet sensors. Of which, 276 (25.4%) of these collisions were extracted for further analysis as they involved pairs of players with head impact sensors and video-verified collisions. Overall, the median linear head acceleration experienced by players was 13.9 (14.7) g, the median rotational velocity was 12.5 (8.8) rad/s, and the median rotational acceleration was 740.2 (1095.3) rad/s^2^.

When the impacts were examined as a whole, there were no significant differences in linear acceleration (*F*_1,447_ = 0.37, *p* = .54), rotational velocity (*F*_1,453_ = 0.42, *p* = .52), or rotational acceleration (*F*_1,454_ = 1.02, *p* = .31) between striking and struck players (Table 1). There were also no significant interactions between player involvement and impact type for linear acceleration (*F*_1,104_ = 3.22, *p* = .08), rotational velocity (*F*_1,274_ = 0.03, *p* = .86), or rotational acceleration (*F*_1,140_ = 0.20, *p *= .66, Table 1). There was a significant interaction between player involvement and impact location for measures of rotational acceleration (*F*_3,524_ = 4.36, *p* = .005) but not linear acceleration (*F*_3,521_ = 1.13, *p* = .34, Table 1) or rotational velocity (*F*_3,507_ = 1.04, *p* = .38). Post hoc testing revealed that collisions to the back of the head had larger rotational accelerations than collisions to the front (*t*_*523*_ = 2.99, *p* = .02) and left (*t*_*515*_ = 3.50, *p* = .003) of the head for the striking player.

**Figure 3:**
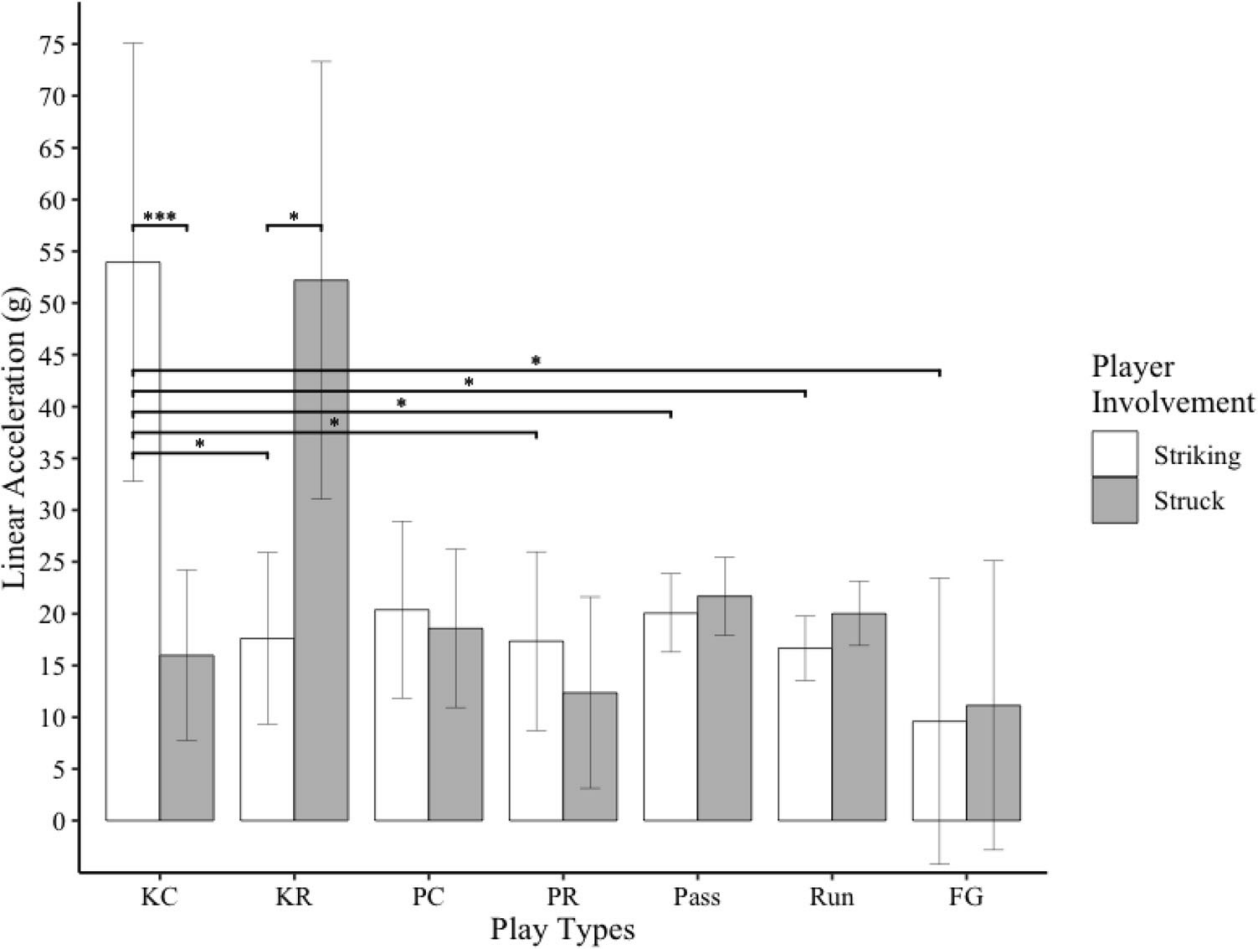
Bar graph of linear head acceleration for striking and struck players during different types of football plays. Error bars indicate 95% confidence intervals. * indicates *p* < .05. *** indicates *p* = .001. KC = kickoff cover; KR = kickoff return, PC = punt cover; PR = punt return; and FG = field goal.

**Figure 4:**
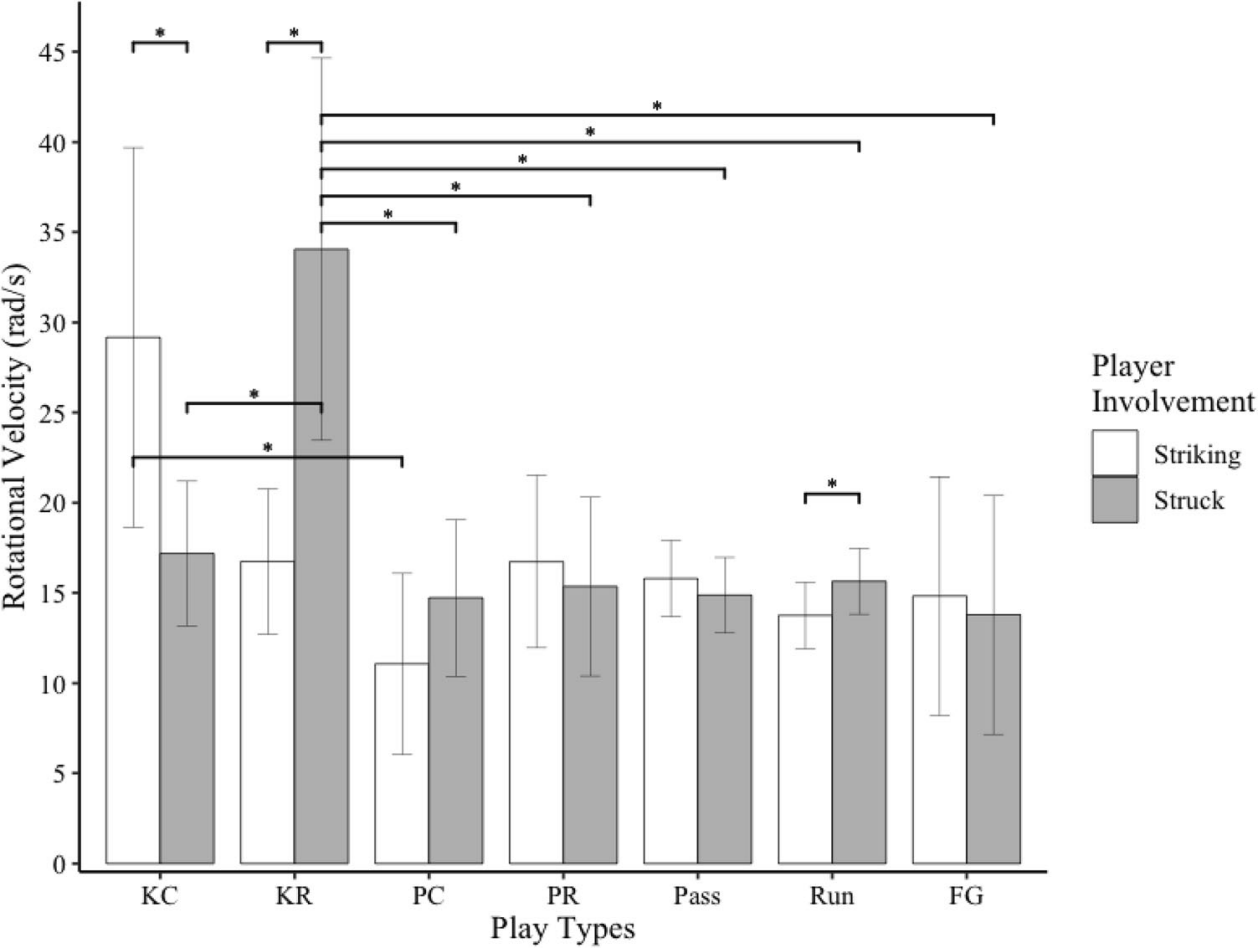
Bar graph of rotational head velocity for striking and struck players during different types of football plays. Error bars indicate 95% confidence intervals. * indicates *p* < .05. *** indicates *p* = .001. KC = kickoff cover. KR = kickoff return, PC = punt cover; PR = punt return; and FG = field goal.

**Figure 5:**
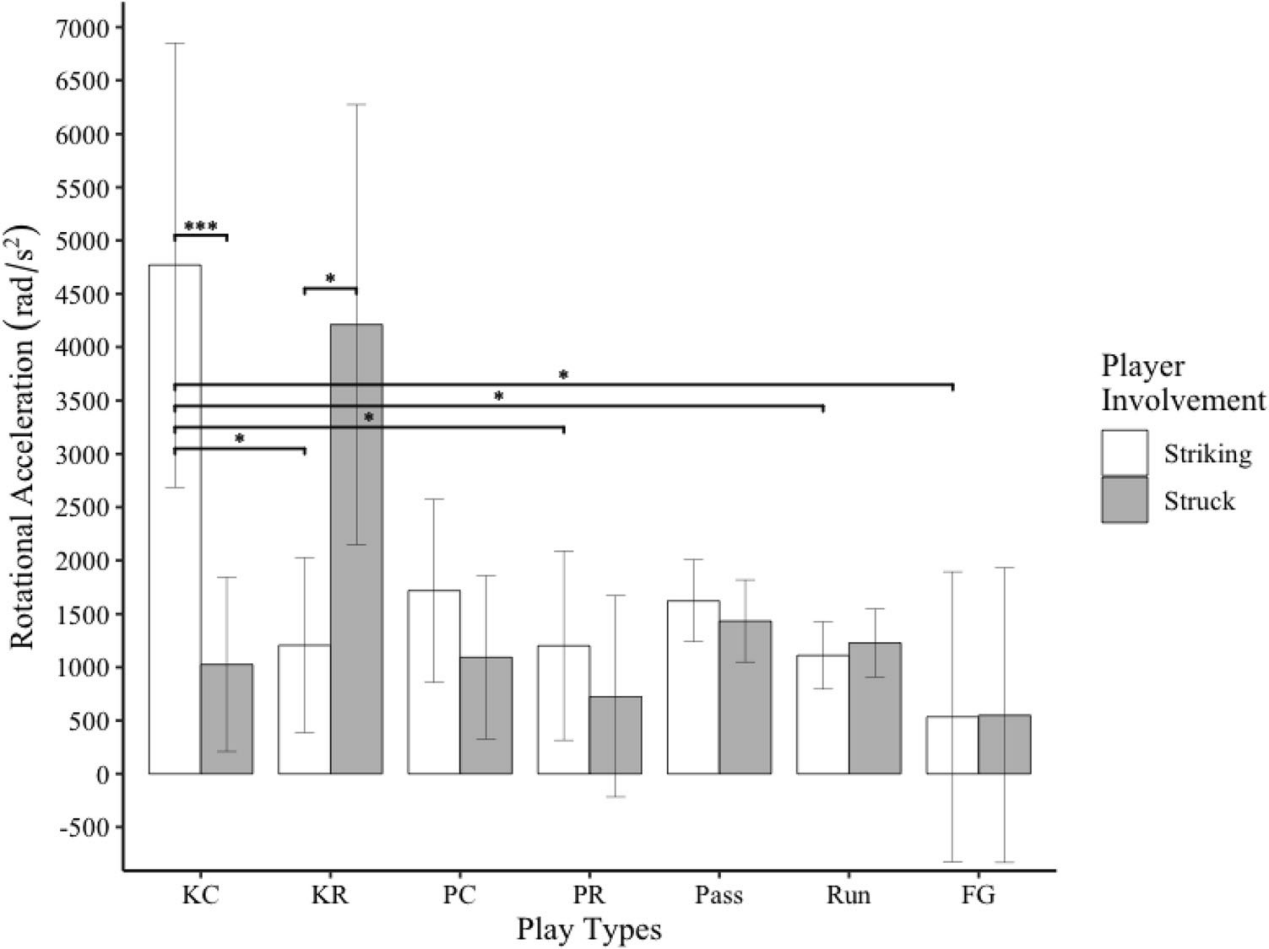
Bar graph of rotational head acceleration for striking and struck players during different types of football plays. Error bars indicate 95% confidence intervals. * indicates *p* < .05. *** indicates *p* = .001. KC = kickoff cover; KR = kickoff return, PC = punt cover; PR = punt return; and FG = field goal.

**Table 1:** Mean linear acceleration, rotational velocity, and rotational accelerations of Canadian Varsity Football Player’s heads for player involvement across entire study, and the interactions with impact type and impact location.

	Linear acceleration (g)				Rotational velocity (rad/s)				Rotational acceleration (rad/s^2^)			
	Mean	95% CI		*p*	Mean	95% CI		*p*	Mean	95% CI		*p*
		L	U			L	U			L	U	
*Player Involvement*												
Striking^a^	22.2	17.7	26.7	(Ref)	16.9	14.4	19.3	(Ref)	1737.7	1284.4	2191.1	(Ref)
Struck	21.7	17.3	26.1	.85	17.9	15.5	20.4	.46	1466.8	1022.6	1911.0	.36
*Impact Type*												
*Block*												
Striking^a^	22.6	18.1	27.1	(Ref)	15.8	13.4	18.2	(Ref)	1663.5	1212.2	2114.7	(Ref)
Struck	19.2	14.7	23.8	.24	17.0	14.6	19.5	.38	1319.8	869.0	1770.7	.23
*Tackle*												
Striking^a^	21.9	16.2	27.6	(Ref)	17.9	14.8	21.1	(Ref)	1812.0	1232.7	2391.4	(Ref)
Struck	24.2	18.6	29.7	.53	18.9	15.8	21.9	.63	1613.7	1051.2	2176.3	.60
*Impact Location*												
*Striking*												
Back^a^	18.5	12.4	24.6	(Ref)	21.7	18.6	24.8	(Ref)	2340.2	1726.1	2954.2	(Ref)
Front	20.8	16.0	25.7	.81	12.1	9.5	14.6	< .001^b^	1553.0	1062.7	2043.4	.02^b^
Left	23.5	18.0	28.9	.33	16.4	13.6	19.2	< .001^b^	1319.9	773.4	1866.5	.003^b^
Right	26.1	20.6	31.6	.06	17.3	14.5	20.1	.008	1737.9	1188.8	2286.9	.20
*Struck*												
Back^a^	13.4	7.6	19.2	(Ref)	20.8	17.9	23.8	(Ref)	1159.5	580.6	1738.4	(Ref)
Front	22.2	17.3	27.1	.003^b^	13.9	11.2	16.5	< .001^b^	1601.0	1110.6	2091.4	.29
Left	24.4	19.1	29.7	< .001^b^	18.2	15.4	21.0	.12	1348.3	818.2	1878.4	.89
Right	26.8	21.5	32.2	< .001^b^	18.8	16.0	21.6	.38	1758.4	1222.9	2293.8	.14

^a^Denotes the reference category (Ref) used for post hoc testing

^b^Significantly different than reference category

There was a significant interaction between player involvement and play type for measures of linear acceleration (*F*_6,265_ = 3.23, *p* = .004), rotational velocity (*F*_6,330_ = 2.76, *p* = .01), and rotational acceleration (*F*_6,280_ = 3.10, *p *= .006). Striking players experienced significantly greater linear head accelerations during kickoff plays than field goal (*t*_*303*_ = 3.48, *p* = .01), kick return (*t*_*265*_ = 3.19, *p* = .03), pass (*t*_*251*_ = 3.12, *p* = .03), punt return (*t*_*248*_ = 3.20, *p* = .03), and run (*t*_*247*_ = 3.46, *p* = .01) plays. Struck players did not experience significantly different linear accelerations between any play types (*p* > .05). Striking players experienced greater linear accelerations than struck players during kickoff plays (*t*_*267*_ = 3.30, *p* = .001). Struck players experienced greater linear accelerations than striking players during kick return plays (*t*_*274*_ = 3.01, *p* = .003, Fig. 3).

Striking players experienced significantly greater rotational head velocities during kickoff plays than punt cover plays (*t*_*237*_ = 3.13, *p* = .03). Struck players experienced significantly greater rotational velocities during kick return plays than field goal (*t*_*264*_ = 3.22, *p* = .02), punt cover (*t*_*232*_ = 3.40, *p* = .01), punt return (*t*_*236*_ = 3.24, *p* = .02), run (*t*_*224*_ = 3.42, *p* = .01), and pass (*t*_*231*_ = 3.53, *p* = .009) plays. Struck players also experienced significantly greater rotational velocities during kickoff plays than kick return plays (*t*_*241*_ = 2.98, *p* = .049). Striking players experienced greater rotational velocities than struck players during kickoff plays (*t*_*242*_ = 2.11, *p* = .04). Struck players experienced greater rotational velocities than striking players during kick return (*t*_*248*_ = 3.02, *p* = .003) and run (*t*_*365*_ = 2.02, *p* = .04, Fig. 4) plays.

Striking players experienced significantly greater rotational head accelerations during kickoff plays than field goal (*t*_*328*_ = 3.38, *p* = .01), kick return (*t*_*290*_ = 3.18, *p* = .03), punt return (*t*_*274*_ = 3.15, *p* = .03), and run (*t*_*272*_ = 3.45, *p* = .01) plays. Struck players did not experience significantly different rotational accelerations between any play types. Striking players experienced greater rotational head accelerations than struck players during kickoff plays (*t*_*291*_ = 3.30, *p* = .001). Struck players experienced greater rotational head accelerations than striking players during kick return plays (*t*_*300*_ = 2.67, *p* = .008, Fig. 5).

## Discussion

The purpose of this study was to characterize kinematic head impact magnitudes between striking and struck Canadian university football players. We hypothesized that struck players would experience higher head impact magnitudes than striking players, tackling collisions would result in larger head impact magnitudes than blocking collisions, head impact magnitudes would vary by location on the head, and that special teams plays would experience higher head impact magnitudes than offensive or defensive plays. In contrast with our hypothesis, we did not observe any statistically significant differences in the magnitudes of linear acceleration, rotational velocity or rotational acceleration between striking and struck Canadian university football players when all impact and play types and locations were collapsed. Similarly, in terms of impact type, we did not observe any statistically significant differences in the magnitudes of linear acceleration, rotational velocity or rotational acceleration, for both striking and struck players, between blocking and tackling. Striking players experienced greater rotational accelerations for impacts to the back of the head than the front of the head. Kickoff plays exhibited greater linear and rotational head accelerations than most other plays for the striking player. Finally, kickoff plays exhibited significantly larger linear and rotational head accelerations for striking players than struck players, while kick return plays exhibited significantly larger linear and rotational head accelerations for struck players than striking players.

The median linear and rotational head accelerations for striking and struck players reported in this study are lower than measurements in other similar studies.^8^,^11^,^12^,^30^,^31^ Four other studies have measured head impact magnitudes in Canadian university football players using the GFT head impact sensor.^8^,^11^,^12^,^31^ However, only one of them used a location-dependent algorithm to calculate centre of mass impact magnitudes from the helmet shell measurements^8^ which reduces the mean absolute percent error of peak linear and rotational accelerations measurements from 50% to less than 10%. The other studies report raw measurements. The study that used the correction algorithm reported average game impact magnitudes of 21.53 *g* and 1846.4 rad/s^2^, which are comparable to the measurements from our study. The research team that recreated professional level impacts in a laboratory setting using instrumented test dummies measured significantly higher linear and rotational accelerations in the striking (56.1 *g*, 3983 rad/s^2^) and struck (89.4 *g*, 6272 rad/s^2^) players.^45^,^46^ However, the majority of these impacts resulted in concussion in the struck players, whereas none of the impacts measured in the current study resulted in concussions. Furthermore, their impacts were measured from professional athletes so are not generalizable to university football athletes. One study of American university football players^30^ measured slightly higher linear and comparable rotational accelerations in the striking (24.5 *g*, 1401 rad/s^2^) and struck (25.1 *g*, 1502 rad/s^2^) players than those measured in our study. However, they did not include offensive or defensive linemen in their data set. This is important since linemen have lower magnitude impacts than other positional groups,^10^,^29^,^40^ as well as a lower number of extreme impacts (impacts greater than the 95th percentile of the data set) per 1000 impacts.^8^,^10^,^15^,^16^ The addition of linemen to our study sample likely increased the number of low magnitude impacts, thereby decreasing the average magnitudes of measured linear and rotational head accelerations. Finally, a study examining differences in play types measured similar linear (25.2 *g*) and rotational accelerations (1442 rad/s^2^) in special teams plays^34^ than the special teams plays measured in our study.

Previous research has observed greater rotational head accelerations in the struck player than the striking player, and no differences in linear acceleration.^3^,^30^ While our data did not exhibit any statistically significant differences between striking and struck players for either linear or rotational head impact parameters, the confidence intervals for the struck player are almost twice as large as the striking player. This dispersion of data implies that some of the impacts in the struck players were higher magnitude than the striking player. Furthermore, a recent study of professional football collisions resulting in concussion measured higher helmet velocities in the injured player than the non-injured player.^3^ Previous studies have not reported angular velocities for striking and struck players. We observed that the trends for angular velocity and angular acceleration were similar.

While this study is similar in design and player cohort to a study examining striking and struck player head impact magnitudes in American college football,^30^ an important distinction must be made. As is pointed out in their study,^30^ head impact data was only collected from one player for each collision. Thus, the impact magnitudes may have differed for the striking and struck players as they were collected from different collisions. Our study evaluated head impact magnitudes between striking and struck players from the same collision. Accordingly, we were able to draw meaningful comparisons between striking and struck players since they were based on the same collision.

Our hypothesis that tackling collisions would result in larger head impact magnitudes than blocking collisions was not supported. However, we noted blocking styles differed depending upon the play type. In offensive and defensive plays, linemen or running backs engaged with defensive players in close quarters to prevent them from reaching the ball carrier. Defensive players had to react to the play, allowing offensive players to position themselves between the defensive player and the ball carrier to block them. In special teams plays, the play was more dispersed across the field due to the field position change from kicking the ball. Additionally, linemen are not usually involved in special teams plays. Accordingly, there were larger closing distances between faster players, which has been attributed to larger head impact magnitudes.^34^ Taken together, there may be a larger difference between blocking and tackling collisions than what we measured. Additional data is required to investigate this phenomenon.

Striking players experienced greater rotational accelerations for impacts to the back of the head than the front of the head. This can be explained by the striking player’s fast forwards motion of the head when they contact an opponent’s body, but do not engage their own helmet. The forwards motion often measures as an impact location to the back of the head due to the sudden peak linear acceleration measured by the accelerometer in the anterior direction.^30^

Special teams plays have been identified as higher risk, with higher linear and rotational head accelerations measured in collisions with larger closing distances.^34^ Our measurements indicate significantly increased linear and rotational head accelerations on special teams plays compared to pass and run plays on offense and defense, specifically during kickoff and kick return collisions. In the Ivy League of the National Collegiate Athletic Association, kickoffs accounted for 6% of all plays but 21% of concussions.^48^ Accordingly, the kickoff has been highlighted as one of the most dangerous plays in American football. We observed linear accelerations for this play that were twice as large as any other play type, and triple as large for rotational accelerations as any other play type, supporting the concept that Canadian kickoff plays are high risk for participating athletes. The kickoff in American football has undergone rule changes in the recent past. These include the removal of three person “wedges” on the kick return team (three players link arms to form a barrier between other players and the ball carrier), restricting the kickoff team to a five yard run to the line of scrimmage, and moving the line of scrimmage forward to encourage more touchbacks (when the ball is kicked into the opposing team’s end zone and play is stopped).^39^ While there have been ongoing changes to kickoff rules in the American game,^39^,^48^ it is apparent that similar changes should be considered in the Canadian game, in order to reduce the severity of head impacts for participating players.

Rule changes have had varied results in collegiate football player head impact reduction.^36^ Eliminating two practices a day during the preseason increased overall preseason head impact burden^42^ while limiting the number of preseason practices had team-dependent differences in overall head impact burden.^36^,^41^ However, reducing the number of minutes participating in specific high risk drills greatly reduced overall head impact exposure for collegiate football players.^2^ These rule changes are focused on practice structure that can be enforced by coaching staff. The results from our study indicate that kickoff and kick return plays experience significantly higher linear and rotational head accelerations than other play types. Accordingly, rule changes to the game play itself could help reduce the number and severity of these impacts, as well as coaching staff enforcing behavior modifications during practices to encourage the removal of the head from collisions.^36^,^43^

This study does not come without limitations. One team only had a subset of players instrumented with accelerometers while the other team had all players instrumented. Thus, not all impacts between players were measured. Accordingly the measurements made in this study are not representative of an entire Canadian university football game; however, we believe they are still comparable due to similarities in magnitudes of head impacts with other studies.^30^,^34^ This study only measured head impacts from players on two teams in a single game on each of two different seasons. Different coaching schemes influence head impact exposures,^29^ so the results of this study may not be generalizable to other teams of different coaching styles. This study used a linear acceleration threshold of 15 *g* to prevent recording accelerations from normal activities,^33^ which is consistent with best practices.^26^ Other studies have used a 10 *g* recording threshold,^4^,^15^,^30^ which increases the number of measured head impacts and decreases the average magnitude of the impacts. The sensor used in this study has been validated in laboratory measurements,^9^ however it has had mixed results with other testing methodologies,^1^,^25^ and has not been evaluated in on-field measurements.

While no differences between striking and struck players during tackling and blocking were measured in this study, we did observe significant differences for kickoff plays that are particularly meaningful. Linear head accelerations for kickoff plays were double that of other special teams, offensive, and defensive plays and rotational head accelerations were triple. This suggests that rule changes around kickoff plays, as implemented in American college football, would decrease head impact exposure of Canadian university football athletes and make the game safer.
